# Emission Impacts from Sustainable Aviation Fuel Blends via Engine Plume Measurements and Predictive Modeling at the Airport Scale

**DOI:** 10.1021/acs.energyfuels.5c05413

**Published:** 2026-02-23

**Authors:** Steffen Schmitt, Benedict Enderle, Tobias Schripp, Tobias Grein, Nina Gaiser, Sabrina T. K. Jensen, Peter W. Holm, Markus Köhler

**Affiliations:** † Institute of Combustion Technology, German Aerospace Center (DLR), 70569 Stuttgart, Germany; § Ko̷benhavns Lufthavne A/S, 2770 Kastrup, Denmark

## Abstract

Sustainable aviation fuels (SAFs) are receiving increasingly high attention by science, industry, and politics as they are considered an effective tool to reduce the impact of aviation on the climate system and air quality. While numerous experimental studies on the effects of SAFs were performed, these are often limited to specific test scenarios or engine measurements. This work combines predictions based on the DLR SimFuel platform with field data from the first campaign to investigate a 34% hydrotreated esters and fatty acids (HEFA) SAF blend under real-world operating conditions during regular passenger flights. For this purpose, an Airbus A320-251N flying between Copenhagen and Arlanda was fueled with conventional Jet A-1 for 30 flights during 1 week and with a 34% HEFA SAF blend for 85 flights in 2 weeks. The corresponding exhaust gas plumes during taxiing were analyzed by the DLR mobile lab. Equipped with state-of-the-art instruments, this analysis contains total and non-volatile particle number concentrations and size distribution, gas analytics (CO_2_ and NO_*x*_), and weather parameters. The results confirm the beneficial effects of SAF usage toward the air quality by reducing total particle emissions by about 10% and non-volatile particle emissions by about 40%. Also, this data set obtained under real-world conditions provides a valuable basis for model development and validation.

## Graphical abstract

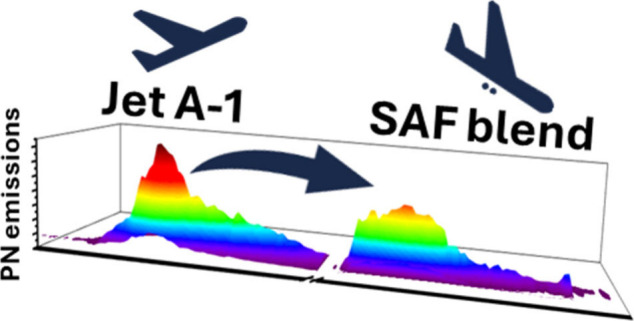

## 1 Introduction

Global deployment of sustainable aviation fuels (SAFs) has entered a ramp-up phase in recent years, driven by initiatives like the U.S. SAF Grand Challenge and the EU’s ReFuelEU SAF blending mandate. SAF is a critical factor in achieving net-zero CO_2_ emissions in aviation,^1–3^ with the added potential to reduce other pollutants emitted by jet engines.^4^ Numerous studies have explored the impact of jet fuel composition on emissions.^5–10^ Both test rig^11,12^ and on-wing studies^13–15^ have demonstrated that reducing soot-precursor compounds in fuels leads to lower emissions of non-volatile particles, both on the ground and in flight. In particular, reducing the levels of aromatics and other unsaturated compounds, correlated with higher hydrogen content, results in reduced soot emissions.^10,16^ Current regulations limit the SAF blending ratio to 50% (sustainable component/fossil jet fuel) and require a minimum of 8.0 vol % aromatics according to ASTM D1655. However, it has to be considered that emissions are not only depending on the aromatics content as shown by the systematic investigations of the ECLIF measurement campaign^17^ where the H/C ratio was found to be a better indicator for soot emission.

Aircraft emissions are the results of a complex interplay between engine technology and combustion kinetics, meaning both aspects have to be investigated to get the overall picture. Model-based approaches as computational fluid dynamics (CFD) simulations are a powerful tool to shed light on this interplay, but also rely on suitable kinetic mechanisms. Since jet fuels are usually complex mixtures and a detailed description thus would be numerically challenging, surrogate strategies^18,19^ as for instance employed in the DLR Concise^20^ mechanism are a good compromise between complexity and computational cost. This mechanism considers a broad variety of n-/iso-/cycloparaffins as well as mono/cyclo-aromatics while still being sufficiently small to be used in complex combustion systems.

Bringing this research into real-world applications requires flexible, data-driven approaches such as the DLR SimFuel^21^ platform, which is based on over 20 000 fuel property data sets. Such approaches enable the analysis of diverse scenarios, e.g., varying SAF usage, in a complex operational context. To ensure the predictive quality thereof, real-world validation data is necessary. Here, airports are one relevant scenario. Since Hudda et al.^22^ demonstrated that ultrafine particles from the Los Angeles international airport (LAX) are transported into the city and can be measured even among other sources, numerous studies have investigated the local air quality at airports and the impact of airport operations on the nearby urban environment. While some studies focused on individual plumes, others presented measurements of the general transport of ultrafine particles into the surroundings. Measurements at Schiphol airport (Amsterdam) demonstrated an increased UFP exposure against 10–20 nm particles downwind the airport.^23^ Mobile measurements at the Berlin airport (BER) revealed that the emission cluster of the airport could be identified at a distance of 7 km downwind the terminal.^24^ Even a vertical emission profile of an airport has been measured via a fixed-wing drone at BER.^25^ Measurements at Stuttgart airport revealed significant changes in the airport emission during the pandemic.^26^

In 2011, Mazaheri et al.^27^ analyzed plumes from several different jet engines at Brisbane airport and produced a corresponding emission inventory. In 2014, Moore et al.^28^ conducted a monitoring campaign near LAX, identifying 275 plumes from various aircraft at a distance of approximately 400 m from the runway. By measuring particle and gaseous concentrations, they calculated emission indices and categorized them by engine type. One notable finding was the relatively similar non-volatile particle number emission index for lean-burn engines (1.95 × 10^15^ #/kg, GE GEnx engines, 2 plumes) compared to conventional engine types. This study demonstrated that emission indices from jet engines can be accurately quantified from a considerable distance using advanced instruments, suggesting that changes in emissions due to fuel composition could also be detected. However, it is important to account for numerous factors beyond fuel composition that can affect jet engine emissions, including ambient conditions (wind and temperature), engine power settings, engine type, and maintenance.^29^ Therefore, a successful monitoring campaign would require a reference fuel tested on the same aircraft under identical conditions.

This study presents a unique approach for assessing non-CO_2_ emission mitigation using a sustainable aviation fuel (SAF) blend in a regular Airbus A320-251N aircraft equipped with CFM LEAP-1A26 engines on the ground. Unlike standard engine emission tests, plume monitoring was conducted using a stationary setup that was situated near a terminal gate of Copenhagen airport (CPH). During departures and arrivals, the aircraft’s plume was measured using fast-scanning instruments in the monitoring station. To minimize influencing factors, the aircraft followed a dedicated flight route between Stockholm/Arlanda (ARN) and CPH, with fuel sourced exclusively from Stockholm. After conducting 30 flights with regular Jet A-1 fuel, the aircraft was then operated with a 34% HEFA SAF blend for 85 flights. The DLR SimFuel platform was used to classify the employed fuels and derive predictions on the emission behavior. A statistical comparison of the measured and predicted particle emission characteristics was performed for the different fuels to assess the potential for particle reduction under regular operating conditions.

## 2 Materials and Methods

The following section describes the DLR mobile lab (section 2.1) employed to measure exhaust gas plumes of the aircraft SE-ROU during passenger flights (section 2.3). The obtained data was evaluated (section 2.2) and further interpreted with the help of the SimFuel platform (section 2.4).

### 2.1 DLR Mobile Lab

The DLR mobile lab, as described in ref 30, was located in front terminal B of CPH. The apron in this area is used as a staging area for baggage and handling vehicles. Aerosol sampling is carried out via a probe installed on the roof at the rear of the vehicle. The inlet of the probe is located at a height of 3.5 m relative to the ground. Consisting of a sampling head for environmental monitoring (Derenda) with an impactor for PM_10_, directly connected to the impactor is a heating pipe (Hillesheim, 8 mm, 3 m) heated to 35 °C which allows a constant tempering of the aerosol for the measuring devices, independent of the outside temperatures. The tempered aerosol is then transferred to a manifold. The manifold has an internal volume of approximately 4 L and enables the aerosol to be distributed to the various measuring devices.

All measuring devices operated on the manifold continuously record the measurement data at a recording rate of 1 Hz. The particles were measured using the following devices: The particle size distribution was determined using an engine exhaust particle sizer (3090 EEPS, TSI), which records the total particle distribution in the range from 6 to 523 nm. In parallel, a differential mobility spectrometer (DMS500, Cambustion) was operated with a catalytic stripper (CS10, Catalytic Instruments) measuring the particle size distribution for the non-volatile fraction. The electrometer currents of the DMS500 were reset under “zero air” conditions at least once per day to avoid long-term signal drifts.

To assess the particle source and identification, the combustion gases CO_2_ and water (LI-7200, LICOR), and the nitrogen oxides NO and NO_2_ (CLD64, Eco Physics) were recorded in addition to the particle data. Parallel to the sampling, a weather station (MWS55, Reinhardt) was installed on the other side of the vehicle to provide a detailed categorization of the plume data for entanglement with airport data. The station recorded wind direction, wind speed and ambient conditions.

### 2.2 Data Processing

Raw data of individual instruments exhibit temporal shifts due to different computer and instrument response times. Therefore, all experimental data were temporarily aligned by comparing peak positions. Afterward the UFP measurement devices for nvPM_number (DMS500) were corrected for particle losses caused by catalytic strippers by using manufacturer-provided algorithms (Catalytic Instruments) that were verified at the World Calibration Centre for Aerosol Physics (WCCAP). Also, EEPS and DMS500 were tested with polydisperse silver particles against the reference SMPS system of the WCCAP. While a satisfying agreement was found for the EEPS, the necessity for a calibration factor of 0.74 was determined for the DMS500. For the relevant particles in this work (i.e., below 100 nm) this factor was observed to be independent of the particle size which is shown in Figure S1 of the Supporting Information.

The resulting unified and calibrated data set was combined with flight data of the target aircraft SE-ROU (see section 2.2) obtained at flightradar24 (www.flightradar24.com). The data on flightradar24 is based on automatic dependent surveillance–broadcast (ADS-B). Figure 1a represents an aircraft exhaust plume that was assigned according to aircraft movement and wind conditions (see Figure 1b). Corresponding particle size distributions are displayed in Figure S2 of the Supporting Information and exhibit monomodal distributions of UFP up to 20 nm in size.

**Figure 1:**
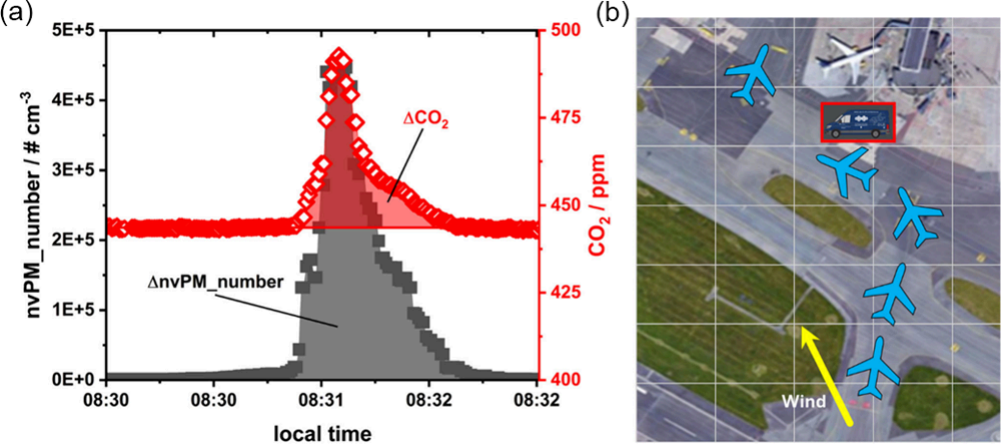
(a) Time-resolved CO_2_ and nvPM_number progression during the target aircraft moving past the DLR mobile lab with ΔCO_2_ and ΔnvPM_number indicated by shaded areas. (b) Aircraft coordinates corresponding to the peak in panel a. The wind direction is indicated by a yellow arrow.

After peak identification and assignment background-corrected integrals were determined for tPM_number, nvPM_number, CO_2_, and NO_*x*_. Please refer to Figure 1a for a graphical illustration of ΔCO_2_ and ΔnvPM_number. Since the dilution of aircraft exhaust gas plumes differ in dependence of several parameters, like weather conditions, aircraft speed, etc., it is necessary to calculate emission indices that refer to an amount of pollutant per kg fuel burned. This follows the approach by Moore et al.^14,28^ and emission indices for nvPM_number (EI nvPM_number), tPM_number (EI tPM_number), and NO_*x*_ [EI­(NO_*x*_)] are calculated as follows:

$$\mathrm{EI nvPM\_number}=\frac{\Delta \mathrm{nvPM\_number}}{\Delta {\mathrm{CO}}_{2}}\cdot \frac{V_{m}}{M\left({\mathrm{CO}}_{2}\right)}\cdot 3160\frac{g}{\mathrm{kg}}$$ 1

$$\mathrm{EI tPM\_number}=\frac{\Delta \mathrm{tPM\_number}}{\Delta {\mathrm{CO}}_{2}}\cdot \frac{V_{m}}{M\left({\mathrm{CO}}_{2}\right)}\cdot 3160\frac{g}{\mathrm{kg}}$$ 2

$$\mathrm{EI}\left({\mathrm{NO}}_{x}\right)=\frac{\Delta {\mathrm{NO}}_{x}}{\Delta {\mathrm{CO}}_{2}}\cdot \frac{M\left({\mathrm{NO}}_{2}\right)}{M\left({\mathrm{CO}}_{2}\right)}\cdot 3160\frac{g}{\mathrm{kg}}$$ 3

where *V*_m_ refers to the molar volume of ideal gas at standard conditions (22.4 L mol^–1^) and *M*(CO_2_) refers to the molar mass of CO_2_ (44.01 g mol^–1^). Instead of M­(NO_*x*_) the molar mass of NO_2_, *M*(NO_2_) (46.005 g mol^–1^), was used in eq 3, since the introduction to the ICAO-EEDB calculates all nitrogen oxides in the sample as if they would be present as NO_2_. In the analyzed events, a strong fluctuation of the NO/NO_2_ ratio with a mean ratio of approximately 2:1 was observed, which is likely to be significantly influenced by ambient conditions (e.g., sun radiation or O_3_ concentrations) not monitored in this work. Nevertheless, the common approximation of 100% NO_2_ was followed in order to achieve comparability to other works and databases. 3160 g kg^–1^ is the mean amount of CO_2_ emitted at a complete combustion of Jet A-1. This factor is dependent on the C/H ratio of the fuel, but the uncertainty introduced by assuming a constant C/H ratio is very small (about 1%^14^) compared to the measurement uncertainty.

For the whole measurement campaign, this analysis was performed for each taxi movement of the target aircraft SE-ROU which is described in the following section 2.3.

### 2.3 Aircraft Operation

During the measurement campaign the target aircraft SE-ROU was operated by Scandinavian Airlines (SAS) flying regular passenger flights mostly between CPH and ARN. The aircraft itself is an Airbus A320-251N (MSN 9262), that has been in service since 03.12.2019, and is equipped with 2x CFM LEAP-1A26 engines. Most important emission characteristics on this engine type according to the ICAO Aircraft Engine Emissions Databank (ICAO-EEDB)^31^ are summarized in Table S1 of the Supporting Information. In the reference phase from January 10th to 16th, 2023 SE-ROU was fueled with Jet A-1 from the regular fuel supply system resulting in ten different batches being employed. During the SAF-blend testing period from January 17th to February 2nd, 2023 the aircraft was operated exclusively on a single, dedicated batch of a 34% HEFA SAF blend. This batch was provided to the aircraft by a separate tank and did not come into contact with the regular Jet A-1 hydrant system to prevent any contaminations with regular Jet A-1. For the same reason, the first flights at the beginning of the SAF blend testing period were neglected for the data interpretation in section 3. Fuel properties and compositions are discussed in the following section.

### 2.4 SimFuel Platform

To evaluate the emission reduction potential of SAF blends and support fuel-specific analysis of the measurement campaign, the DLR software platform SimFuel^21^ was used. The platform features a database of fuel properties, currently comprising approximately 20 000 conventional jet fuels and 500 synthetic fuels and blends. In addition to empirical data, SimFuel includes machine-learning-based models trained on the database to estimate fuel properties from detailed chemical composition^32,33^ or correlations between existing parameters.^34^ An important advantage of the SimFuel platform lies in its extensive coverage of fuel types, engine configurations, and aircraft models. Provided the predictive capability of the model is validated against measurement data, as addressed in later sections, SimFuel enables the analysis of a wide range of operational scenarios that would be impractical to investigate experimentally due to the complexity, cost, and logistical effort involved.

Using fuel supply data from Copenhagen Airport (2020–2021), the minimum SAF blend required to yield detectable changes in aircraft emissions compared to the conventional baseline was estimated. Results indicate that a blend containing 20–30% neat synthetic blending component (SBC) is necessary to produce measurable effects under fully operational campaign conditions.

## 3 Results and Discussion

The following section presents the results of this work. First, the predictions obtained by the DLR SimFuel platform are analyzed and in a second step compared to the real-world emission measurements.

Table 1 summarizes key fuel properties of the conventional Jet A-1 and the HEFA SAF blend used during the measurement campaign. HEFA derived from used cooking oil (UCO) feedstock was selected, as it is currently the predominant SBC product available on the market among the eight approved pathways under ASTM D7566, thereby providing a representative indication of present SAF emission-reduction potential. The SAF blend batch was not tailor-made for the trial but procured through standard commercial purchasing channels from airBP in order to reflect real-world fuel availability as closely as possible. Consequently, detailed information on the conventional fuel component used for blending was not available. Although ASTM D1655, Table 1 permits HEFA SAF blends with up to 50% SBC, the blend used for the trial contained 34% SBC, which is representative of SAF blend products currently available on the market.

**Table 1:** Selected Fuel Properties of Conventional Jet A-1 Batches and SAF Blend Batch

	conventional reference, range (95% CI)^a^	SAF blend, mean (95% CI)^a^
composition	100% Jet A-1	34% HEFA-SPK, 66% Jet A-1
total aromatics % (vol/vol)	17.2–19.9 (16.72–20.43)	12.2 (11.82–12.58)
hydrogen content % (kg/kg)	13.80–14.14^b^ (13.74–14.2)	14.34^c^ (14.28–14.40)
sulfur % (kg/kg)	0.050–0.191 (0.043–0.218)	0.046 (0.04–0.05)
specific energy (MJ/kg)	43.296–43.321 (43.286–43.332)	43.539 (43.528–43.550)

a 95% confidence interval based on repeatability as defined in the respective standard.

b ASTM D3343 correlation.

c ASTM D7171 measurement (^1^H NMR).

Due to the complexity of the fueling infrastructure at a major airport such as Arlanda, tracking individual fuel batches from import to uplift is not possible. Consequently, only the observed range of properties is reported for conventional fuels. Aromatics content for these fuels was obtained from certificates of analysis (CoA) issued for each batch imported into the airport system. Hydrogen content for the HEFA SAF blend was determined according to ASTM D7171 (^1^H NMR), while for the conventional fuels, hydrogen content was estimated using the ASTM D3343 method based on CoA-derived parameters.

The aromatics content of the conventional fuels falls within the typical range, whereas the SAF blend exhibits significantly lower values, consistent with the near-zero aromatics content of HEFA-SPK blending components.

Figure 2 compares hydrogen content of the fuels used during the campaign with values from the SimFuel database, covering 27 representative conventional fuels and 15 SAF blends. Most conventional batches fall below the database median, and the SAF blend likewise exhibits hydrogen content below the typical range observed for blends in the database.

**Figure 2:**
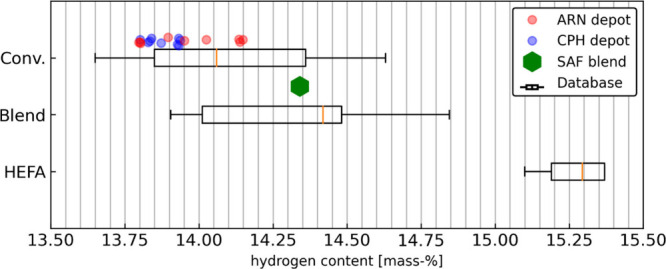
Comparison of hydrogen content in conventional Jet A-1 batches and SAF blend batches with SimFuel database ranges.

To estimate the potential reduction in black carbon emissions attributable to the SAF blend, a Bayesian model was developed based on auxiliary power unit (APU) measurement data from the JETSCREEN^35^ project, which closely replicates real aircraft taxi conditions. The model relates relative black carbon mass concentration to fuel hydrogen content. Figure 2 presents the experimental data, along with the model’s posterior predictive distribution (mean and 95% credible interval). The hydrogen content range of the conventional Jet A-1 batches spans a corresponding range of expected black carbon emissions under model uncertainty (shaded dark blue area).

To further illustrate this, samples from the posterior predictive distribution of the Bayesian model are shown in Figure 3b as boxplots, representing the expected black carbon emissions for the conventional Jet A-1 batches (within the shaded dark blue range) and the SAF blend (aligned with the green vertical line). The predicted emission range for the conventional fuels is clearly higher than for the SAF blend, reflecting both the lower hydrogen content and the greater model uncertainty in this region. Based on the difference between the medians (orange lines), the mean expected reduction in black carbon emissions when using the SAF blend is estimated at 37% with a 95% confidence interval of (36–38%) and *p* = 1 × 10^–5^ from Mann–Whitney *U* test. It should be noted that the modeling approach represents a generalization, as it does not account for specific aircraft or engine types. A comparison with measured data will be presented in subsequent sections to assess the validity of the predictions.

**Figure 3:**
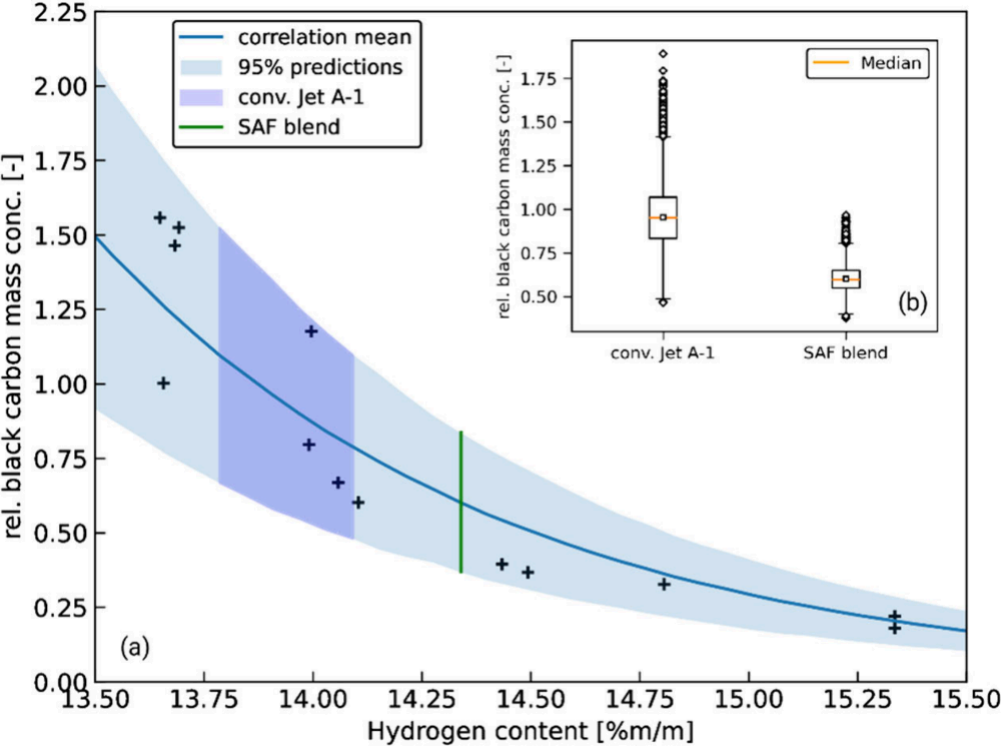
(a) Modeled correlation between fuel hydrogen content and relative black carbon mass concentration using a Bayesian approach. The model is trained on experimental data (black crosses) from a previous measurement campaign under APU-like operating conditions. Shaded areas represent the posterior predictive distribution (mean and 95% credible interval). (b) Distribution of predicted black carbon mass concentrations for the conventional Jet A-1 batches and the SAF blend, derived from the posterior predictive distribution.

Meteorological parameters are of the utmost importance for the interpretation of data. The main wind direction at CPH is southwest, which is why the sampling location north of the taxiway was chosen (see Figure 1b) enabling aircraft plumes to be measured at wind directions between approximately 135° (SE) and 270° (W). The weather conditions were excellent for emission measurements during the campaign with south wind for about 75% of the time. A wind rose plot with data of the weather station operated on the DLR mobile lab (see section 2.1) is displayed in Figure S3 of the Supporting Information. Although this weather station is not as representative and accurate as the weather station operated by the Danish Meteorological Institute at CPH, it is essential to measure the local wind regime at a high frequency of 1 Hz. Since nearby terminal buildings may alter the wind conditions and fast fluctuations of the wind direction, the available 5 min average values publicly may not be sufficient for a detailed analysis.

Especially at times with frequent aircraft and ground vehicle movements, exhaust gas plumes sometimes overlapped (see Figure S4 of the Supporting Information for an exemplary morning). For the further analysis on the influence of the SAF blend, it is necessary to exclude all SE-ROU movements where a further emission source, either another aircraft or ground operation vehicles, contributed significantly to the measured parameters. The resulting analyzed events meeting all quality criteria are summarized in Figure 4 which corresponds to 60 and 70% of the performed SE-ROU flight movements for the reference and test phase, respectively.

**Figure 4:**
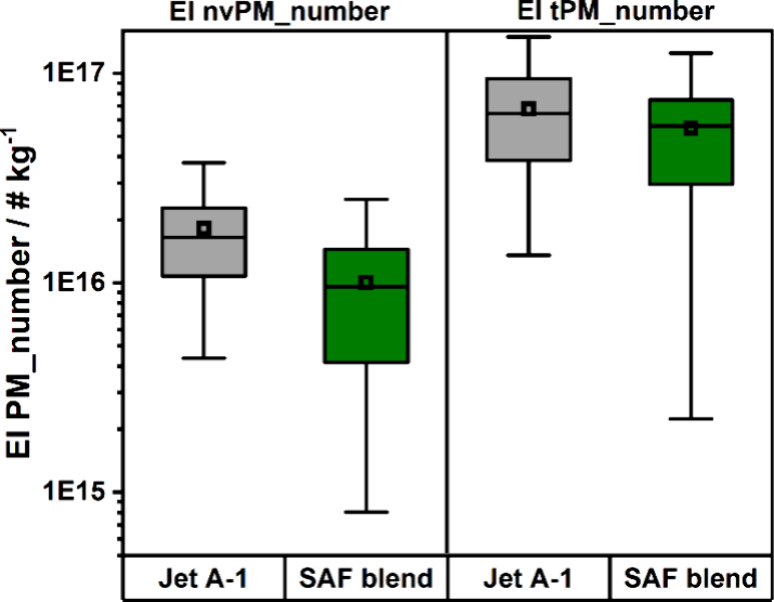
Boxplot of EI nvPM_number and EI tPM_number for all events successfully analyzed during the campaign. The reference phase with Jet A-1 is displayed in black, while the green boxes correspond to using a 34% HEFA-SPK SAF blend.

Note that the spread of measured EI’s for UFP emissions is not only caused by the measurement uncertainty, but also fluctuations in influencing factors like pilot behavior, engine maintenance status, fuel composition and properties (for the Jet A-1 reference phase), or weather conditions. As the operational data of the aircraft engine is not accessible judging the impact of pilot behavior and engine settings is challenging. However, EI­(NO_*x*_) values (see Figure 5) can serve as a rough indicator for the engine thrust setting, since EI­(NO_*x*_) increase monotonically with the thrust level. Similar EI­(NO_*x*_) values of (3.3 ± 1.8) and (4.0 ± 1.0) g/kg were found for reference and test phase, respectively. This is an indication for similar engine setting during taxiing during the two phases of the campaign. In addition to that the obtained values are very similar to the ICAO-EEDB value of 4.63 g/kg^31^ for idle engine conditions.

**Figure 5:**
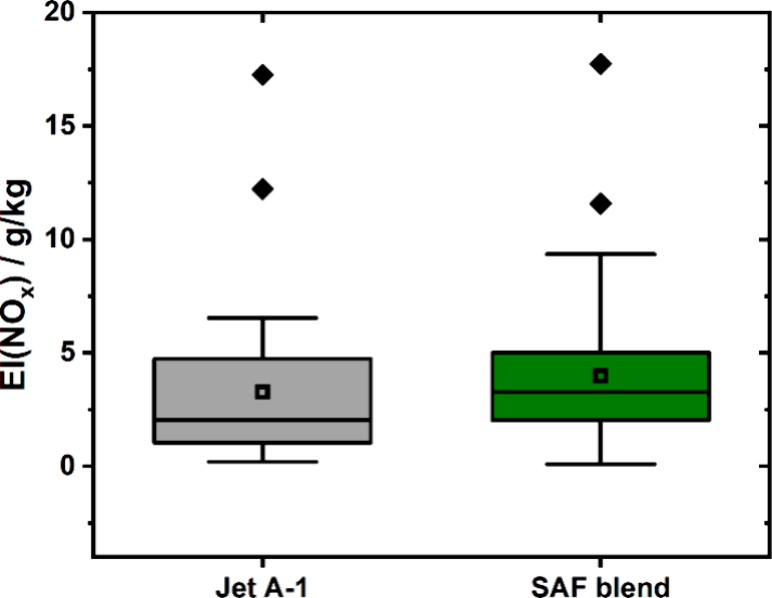
Boxplot of EI­(NO_*x*_) for all events successfully analyzed during the campaign. The reference phase with Jet A-1 is displayed in black, while the green boxes correspond to using a 34% HEFA-SPK SAF blend.

In general, the engine maintenance status can also be expected to alter the engine’s emission characteristics. For instance, Sogut et al.^36^ concluded that degradation of an aircraft engine can lead to a loss in engine efficiency and Chen and Sun found an emission reduction due to on-wing washing.^37^ However, since the campaign was performed with the same aircraft that did not undergo any major overhauls and repairs during the test period, this effect can be considered rather small here. A clear correlation between a meteorological parameter and the resulting emission indices could not be found. This leads to the conclusion, that this does not introduce a bias in the following interpretation. In addition to that reference and test phase experienced similar weather conditions as is shown in Figure S5 of the Supporting Information.

The large impact of the fuel composition on the emissions, and consequently the significant potential for emission reduction, becomes evident upon analysis of the spread of emission indices during the reference phase of Jet A-1. Here, ten different batches were supplied at ARN/CPH during that period and therefore a variety of Jet A-1 fuels was burned resulting in a larger variation of emission indices. The extraordinary large spread of EI tPM_number’s in the Jet A-1 reference might be explained by the strong deviations in sulfur content ranging from 0.05 to 0.187 wt %. A higher sulfur content leads to more sulfur oxidation products, which can increase the ice nucleating potential of soot and enhance the formation of volatile particles.^38^

Although the experimental values exhibit a substantial spread due to the aforementioned factors, a median reduction of 42% (95% CI = 17–62%; *p* = 0.0005) in non-volatile particle emissions and 12% (95% CI = −35–44%; *p* = 0.074) in total particle emissions was observed for EI tPM_number and EI nvPM_number, respectively.

The 95% confidence intervals were estimated using non-parametric bootstrap resampling within the two fuel groups (10 000 resamples). Statistical significance was assessed using the Mann–Whitney *U* test. While the reduction in nvPM emissions is statistically significant, the reduction in tPM emissions shows a large uncertainty range and does not reach statistical significance. Possible reasons for this behavior are discussed toward the end of this section.

It is to be considered that these values were achieved with the same instruments in an identical setup within a time period no longer than a month. Therefore, any systematic uncertainty would not influence the difference between Jet A-1 and SAF blend usage. Recently, Durdina et al.^39^ found a reduction of non-volatile particle mass by about 35% and of about 20% of non-volatile particles numbers when using a similar SAF blend containing 30% HEFA-SPK at idle engine settings corresponding to a taxi movement of the aircraft. Their emission tests were performed on a parked business jet (Cessna Citation 560XL) equipped with a turbofan engine (Pratt & Whitney Canada PW545A)^39^ so it cannot be directly compared to the CFM LEAP-1A26 jet engines investigated here, but the similar observed trends show a consistent picture. To the best of the authors’ knowledge this is the first time that the beneficial effects of SAF have been demonstrated in measurement campaign under realistic operational conditions performing regular passenger flights.

To compare the experimental results with the DLR SimFuel platform’s predictions the pollutant parameters (EI PM_number and relative black carbon mass concentration) were normalized to the corresponding median value of the reference scenario. The resulting box plots are shown in Figure 6.

**Figure 6:**
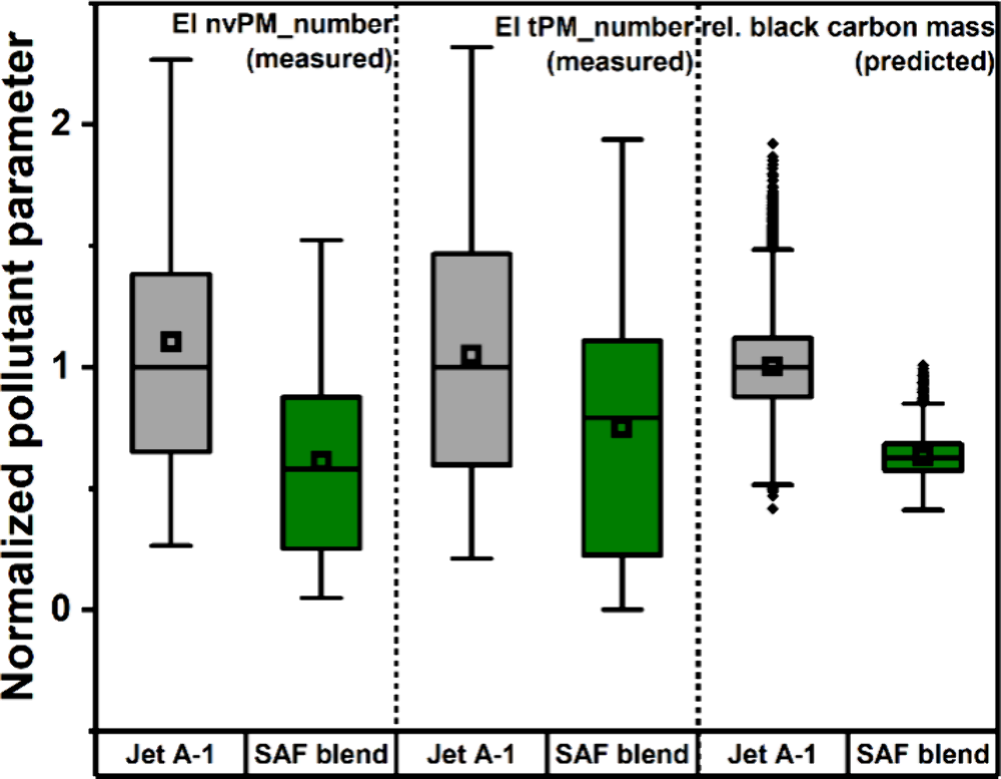
Boxplots of measured emissions indices for both non-volatile and total particle number concentrations and rel. black carbon mass concentrations predicted by the DLR SimFuel platform. For a better comparability, the values were normalized to the median value of the corresponding Jet A-1 reference case.

Here, a good agreement between the measurement results and the predictions of the DLR SimFuel platform is found. Especially for EI nvPM_number and rel. black carbon mass concentration the tendencies are almost identical. For the EI tPM_number the effect of the SAF blend is slightly smaller, which may be caused by the fact, that the share of black carbon in tPM_number is expected to be smaller than in nvPM_number. Recent studies by Ungeheuer et al.^40,41^ and Decker et al.^42^ indicate the importance of aircraft engine oils in the totality of aircraft particle emissions. As engine oil emissions are more likely to be linked to engine technology than fuel composition, the influence of SAF usage is expected to be smaller for tPM number than for nvPM number.

## 4 Conclusion

In order to investigate the impact of SAF usage on the air quality it is necessary to perform studies under operational conditions. However, aircraft emissions at airports depend on several factors apart from the fuel composition which is why the measurement campaign was aimed to minimize further influence factors. Therefore, a single aircraft (A320-NEO) was studied with the same devices at the same sampling location under similar meteorological conditions. The SAF real-world measurements resulted in a decrease of about 40% in non-volatile particle number emissions confirming previous results based on, e.g., test-rig measurements. To the best of the authors’ knowledge this is the first study showing the positive trends while investigating real passenger flights over several weeks.

In addition to that the DLR SimFuel platform was used to predict emission parameters based on fuel compositions and compared to the measurement results. This comparison provides confidence that the DLR SimFuel platform is suitable to predict the influence of SAF usage regarding to real-world emissions. Therefore, it can be considered a powerful tool to analyze future scenarios and develop the most effective strategies of employing limited amounts of SAF.

## Supplementary Material



## Notes

Details on DMS500 calibration, exemplary particle size distributions of aircraft plumes, emission data of CFM LEAP-1A26 engines according to ICAO Aircraft Engine Emissions Databank (EEDB), weather data during measurement campaign, temporal evolution of total particle size distributions, temporal evolution of temperature and humidity during the measurement campaign, and references (PDF)

## ‡

Center for Aviation, ZHAW School of Engineering, CH-8400 Winterthur, Switzerland

## Nomenclature

ADS-B: automatic dependent surveillance–broadcast
APU: auxiliary power unit
CFD: computational fluid dynamics
CoA: certificate of analysis
EEDB: Aircraft Engine Emissions Databank
EI nvPM_number: non-volatile particle number emission index
EI tPM_number: total particle number emission index
HEFA-SPK: hydroprocessed esters and fatty acids synthetic paraffinic kerosine
nvPM_number: non-volatile particle number concentration
SAF: sustainable aviation fuel
SBC: synthetic blending component
tPM_number: total particle number concentration
UFP: ultrafine particle
WCCAP: World Calibration Centre for Aerosol Physics
