# Supplementary material for: Emission Impacts from Sustainable Aviation Fuel Blends via Engine Plume Measurements and Predictive Modeling at the Airport Scale
Source: Energy Fuels. 2026 Feb 23;40(9):4662–9. doi: 10.1021/acs.energyfuels.5c05413 (PMC12969261; doi:10.1021/acs.energyfuels.5c05413)
Supplement: Supplementary file 1 [file ef5c05413_si_001.pdf]

Supporting Information (SI)

to

Emission Impacts from Sustainable Aviation Fuel Blends via Engine Plume Measurements and Predictive Modelling at the Airport Scale

Steffen Schmitt[1], Benedict Enderle[1], Tobias Schripp[†1], Tobias Grein[1], Nina Gaiser[1], Sabrina T.K. Jensen[2], Peter W. Holm[2] and Markus Köhler[1]*

[1]Institute of Combustion Technology, German Aerospace Center (DLR), Pfaffenwaldring 38-40, 70569 Stuttgart, Germany

[2]Københavns Lufthavne A/S, Kastrup, 2770, Denmark

[†]Now at Center for Aviation, ZHAW School of Engineering, Winterthur, CH-8400, Switzerland

Corresponding Author

*Corresponding author email: steffen.schmitt@dlr.de

Table of Contents

1. Details on DMS500 calibration 3

2. Exemplary particle size distributions of aircraft plumes 4

3. Emission data of CFMI LEAP-1A26 engines according to ICAO Aircraft Engine Emissions Databank (EEDB) 5

4. Weather data during measurement campaign 6

5. Temporal evolution of total particle size distributions 7

6. Temporal evolution of temperature and humidity during the measurement campaign...........8

7. References...8

1. Details on DMS500 calibration

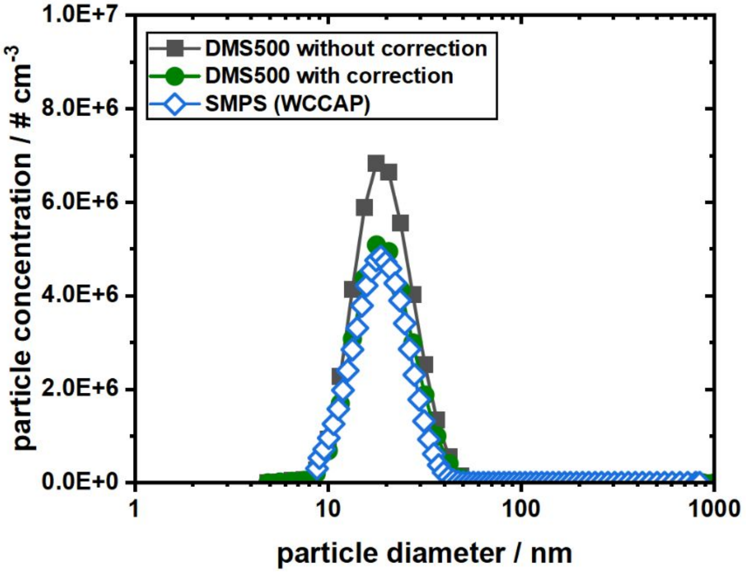

Figure S1. Comparison of particle size distributions measured with DMS500 and the SMPS of the WCCAP. After employing a correction factor (green symbols), DMS500 and SMPS data match well.

2. Exemplary particle size distributions of aircraft plumes

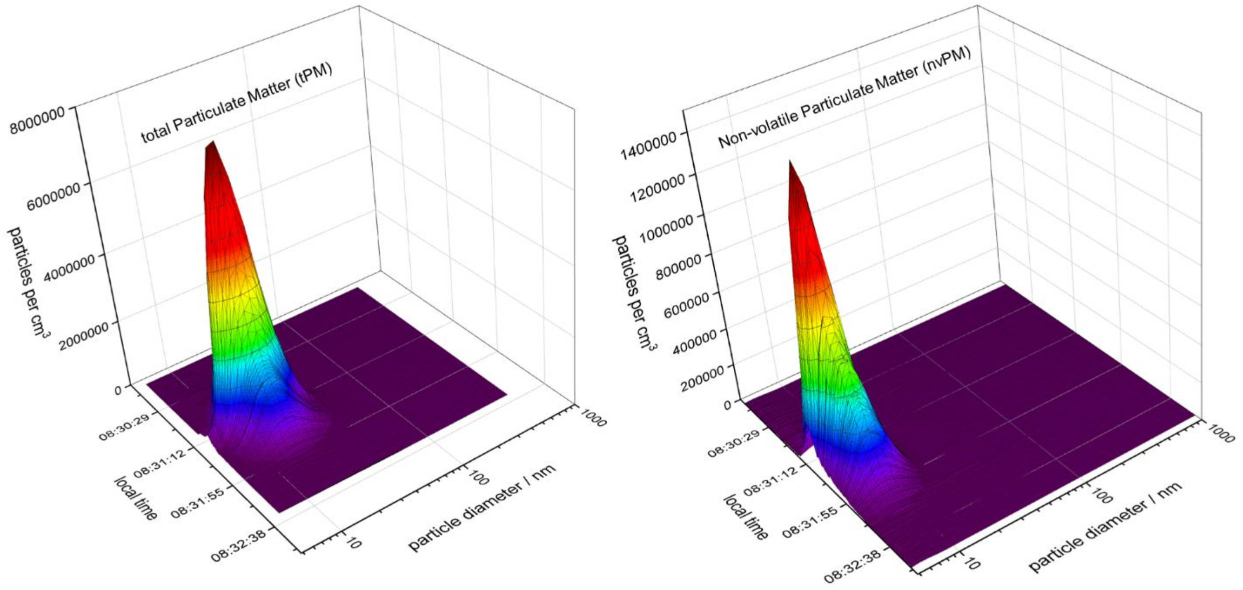

Figure S2. Time-resolved particle size distributions of total (left) and non-volatile (right) particles corresponding to the aircraft exhaust plume discussed in the main manuscript (see Fig. 1).

3. Emission data of CFMI LEAP-1A26 engines according to ICAO Aircraft Engine Emissions Databank (EEDB)

Table S1. Emission data of CFMI LEAP-1A26 engines according to ICAO Engine Exhaust Emissions Databank (EEDB)[1].

	EI nvPM_number [#/kg]	PM [mg/kg]	NO_x [g/kg]
Idle	3.71E+12	0.6	4.63
Approach	6.89E+13	2.0	8.67
Climb off	7.67E+10	1.1	11.16
Take off	8.91E+10	1.4	18.77
Maximum	2.19E+14	7.6	-

4. Weather data during measurement campaign

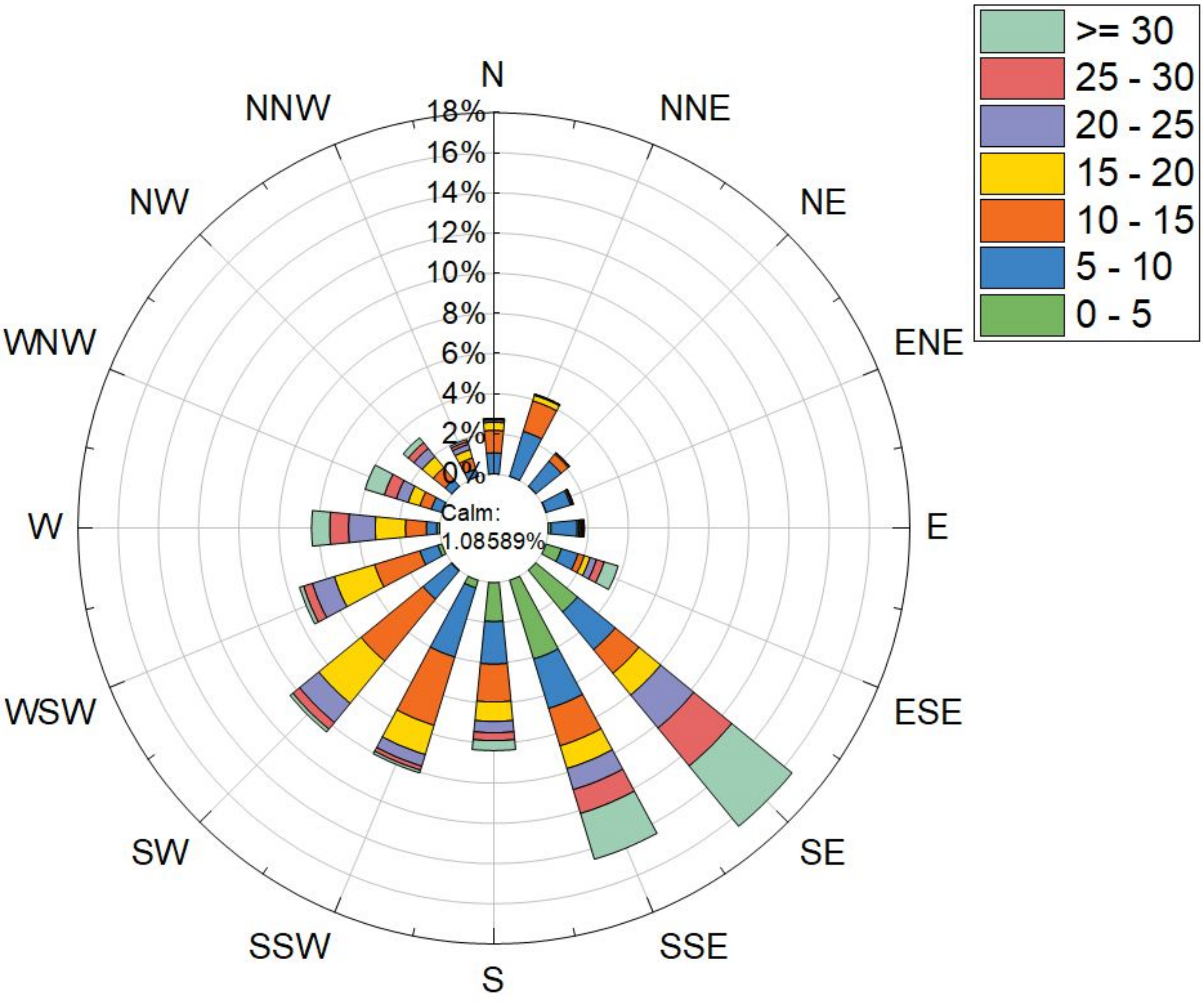

Figure S3. Wind rose measured with the weather station of the DLR mobile lab. The wind speed is displayed in km/h.

5. Temporal evolution of total particle size distributions

Figure S displays a typical morning scenario during the measurement campaign with several aircrafts passing the DLR mobile lab. Three clear and well-defined aircraft exhaust plumes are present between 7:55 and 8:05. At some occasions, the taxiway was used very frequently or nearby aircrafts performed an engine warm-up for several minutes. Therefore, no clear assignment is possible for the events around 8:15. While the peaks at a particles size around 10 nm can be attributed to aircraft emissions, sometimes peaks of particles between 40 and 100 nm appear independently of aircraft movements (e.g. at 7:53). These emissions stem from ground operation vehicles that were often running diesel engines.

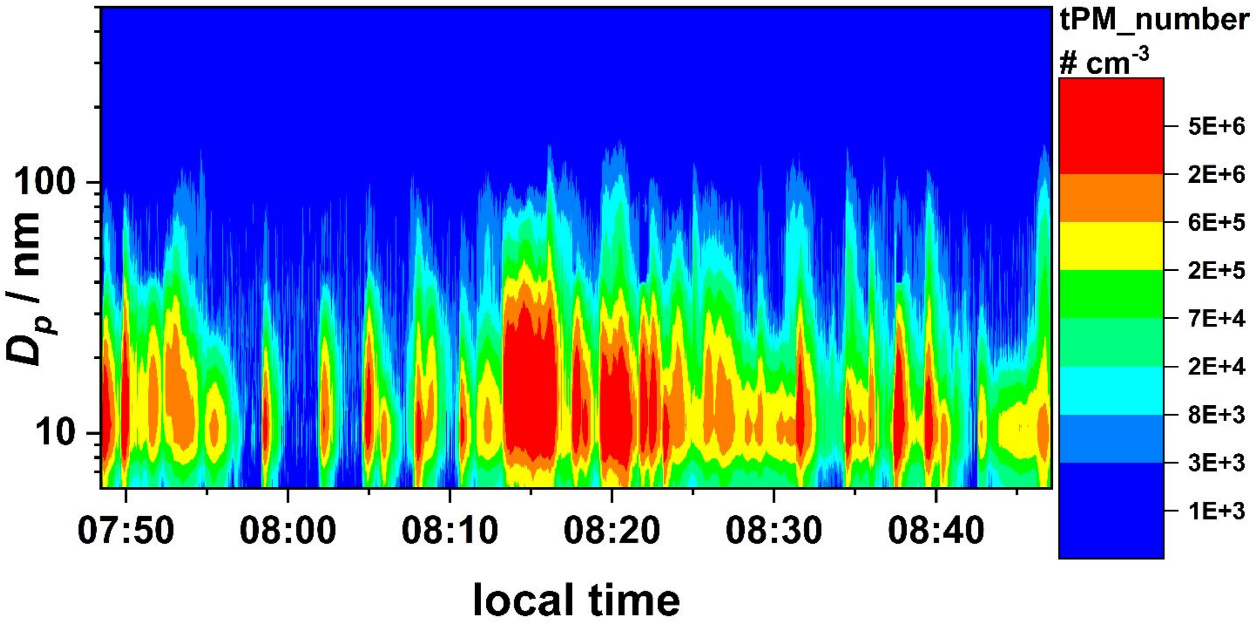

Figure S4. Contour plot displaying the progression of the total particle size distribution with wind coming from SW.

6. Temporal evolution of temperature and humidity during the measurement campaign

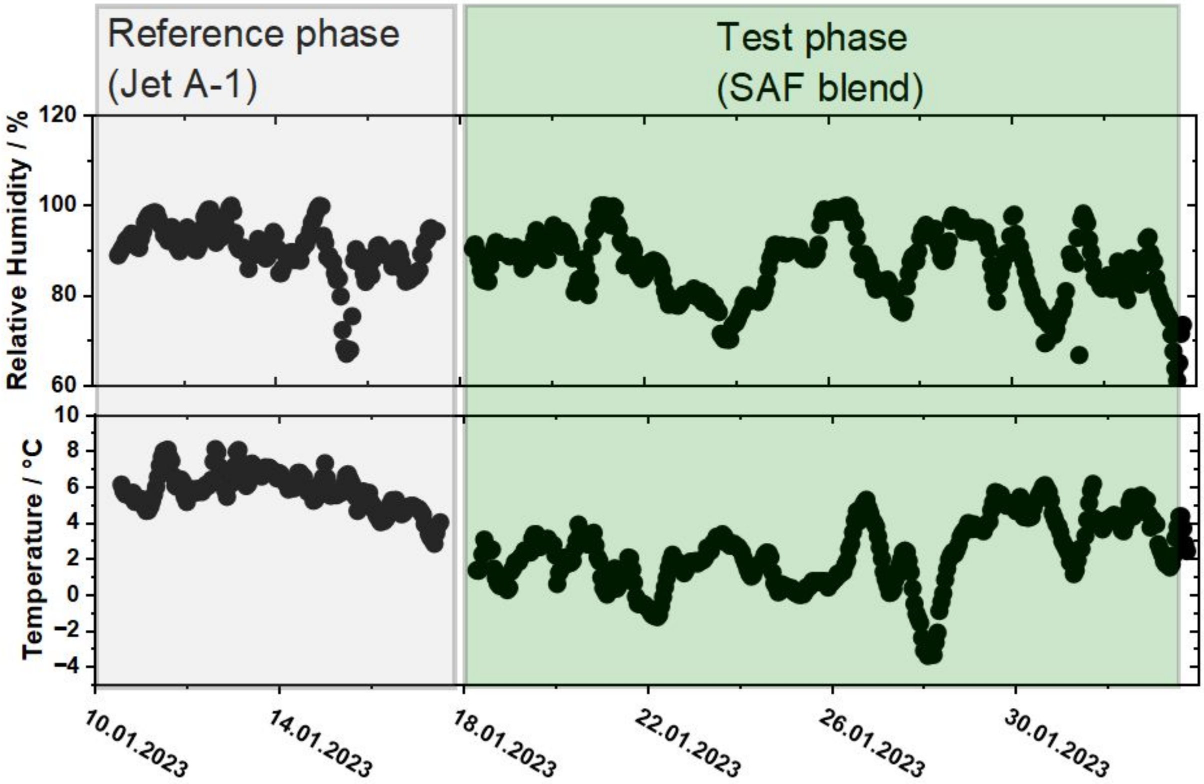

Figure S5. Hourly mean values of relative humidity (top) and temperature bottom during the measurement campaign. The time frame for the reference phase is indicated in grey, while the test period employing SAF blend is indicated in green.

7. References

(1) EASA. *ICAO Aircraft Engine Emissions Databank.* https://www.easa.europa.eu/en/domains/environment/icao-aircraft-engine-emissions-databank#group-easa-downloads (accessed 2025/06/25).
